# Sterile males in a parasitoid wasp with complementary sex determination: from fitness costs to population extinction

**DOI:** 10.1186/s12898-014-0032-6

**Published:** 2015-05-12

**Authors:** Xavier Fauvergue, Anna Chuine, Chloé Vayssade, Alexandra Auguste, Emmanuel Desouhant

**Affiliations:** INRA, UMR 1355 Institut Sophia Agrobiotech, 06903 Sophia Antipolis, France; Université de Nice Sophia Antipolis, UMR Institut Sophia Agrobiotech, 06903 Sophia Antipolis, France; CNRS, UMR 7254 Institut Sophia Agrobiotech, 06903 Sophia Antipolis, France; Université de Lyon, Lyon F-69000, France; Laboratoire de Biométrie et Biologie Evolutive, Universite Lyon 1, CNRS, UMR 5558, F-69622 Villeurbanne, France; Institut Sophia Agrobiotech, 400 Route des Chappes, BP 167, 06903 Sophia-Antipolis Cedex, France

**Keywords:** Sex determination, Extinction vortex, Inbreeding, Inbreeding depression, Diploid males, Hymenoptera, Mate-choice, Population biology

## Abstract

**Background:**

Single-locus complementary sex determination (sl-CSD), which occurs in some insects of the order Hymenoptera, imposes a heavy genetic load that can drive small populations to extinction. The core process in these species is the development of individuals homozygous at the sex-determining locus into unfit diploid males. The risk of extinction of populations with sl-CSD is theoretically much higher if diploid males are viable and capable of mating but sterile, because diploid males then decrease the reproductive output of both their parents and the females with which they mate.

**Results:**

In the parasitoid wasp *Venturia canescens* (Hymenoptera: Ichneumonidae), diploid males resembled their haploid counterparts in most respects, but their mating success was nevertheless lower than that of haploid males, especially when the two types of males were placed in competition. Furthermore, although diploid males transferred viable sperm during copulation, they sired no daughters: the females with which they mated produced only sons, like virgin females. A simulation model combining behavior, genetics and demography demonstrated that for two alternative hypotheses concerning the fertilization success of diploid sperm, the mating success of diploid males strongly affected population dynamics.

**Conclusion:**

The performance of diploid males should be estimated in competitive situations. It is a crucial determinant of the probability of extinction.

**Electronic supplementary material:**

The online version of this article (doi:10.1186/s12898-014-0032-6) contains supplementary material, which is available to authorized users.

## Background

Many intrinsic processes occurring in small populations render these populations prone to extinction. Consequently, the small population paradigm in conservation biology aims to determine the nature and consequences of these processes [1]. Some processes are strictly demographic in nature [2,3], whereas others are genetic [4]. Behavioral processes also affect the fate of small populations [5-8]. Inbreeding perfectly illustrates the feedback between demography, genetics, and behavior. It involves reproduction between genetically related individuals and leads to a decrease in heterozygosity. It has a potential adverse consequence, inbreeding depression, defined as a lower fitness of inbred than of outbred individuals. Inbreeding and inbreeding depression tend to increase in declining populations [9-16]. Inbreeding depression affects life-history traits relating to demographic parameters [17] and may act together with other processes, such as demographic stochasticity, to drive small populations into an extinction vortex [17-23]. However, adapted behavior, such as mate choice and dispersal, may attenuate the adverse genetic consequences of small population size [8,24,25], thereby diminishing the threat of extinction vortices.

The sex determination system of some species of the order Hymenoptera appears to be an interesting paradigm for investigating feedback between demography, genetics and behavior. In many species, single-locus complementary sex determination (sl-CSD) causes a severe form of inbreeding depression. Under sl-CSD, individuals that are diploid and heterozygous for the complementary sex-determiner gene (*csd*) develop into females, whereas individuals that are either haploid and hemizygous or diploid and homozygous develop into males. Haploid males are normal, but diploid males are typically inviable or sterile ([26-28], but for exceptions see [29,30]). Sl-CSD is a form of inbreeding depression, because the expected proportion of unfit diploid males is higher in the offspring of genetically related parents (e.g. 12.5% in the progeny of full siblings). Overdominance is the underlying mechanism [31], as no single allele at the *csd* gene is intrinsically deleterious. The lower fitness of homozygous individuals than of heterozygotes nonetheless generates a severe genetic load, leading to a “diploid male vortex”, with a decrease in population size resulting in a decrease in heterozygosity at the *csd* gene, an increase in the proportion of diploid males, a decrease in population growth rate, and, if severe enough, a further decrease in population size [32]. The diploid male vortex increases the expected risk of extinction in haplodiploids by more an order of magnitude over and above that resulting from inbreeding depression in threatened diploids [32,33]. Hence, observations for complementary sex determination run contrary to the widely held belief that haplodiploid organisms are relatively immune to inbreeding depression [34].

Zayed & Packer [32] predicted that the probability of extinction under single-locus complementary sex determination would be sensitive to the life history and behavior of diploid males. If diploid males are inviable, their production is analogous to a decrease in the survival of immature females (because diploid males develop from fertilized eggs that would normally have developed into females). If diploid males are viable but sterile, their production has two types of effect, over two generations. First, they decrease the production of daughters, for parents sharing an allele of the *csd* gene. Second, they decrease the production of daughters from the females with which they mate (assuming that the eggs fertilized by diploid males are triploid and, therefore, inviable). With the set of parameters used by Zayed & Packer [32], the occurrence of inviable diploid males resulted in a mean extinction rate 30 times higher than that obtained in conditions of demographic stochasticity only, and this increase in extinction rate was two to three times higher still if the diploid males were assumed to be viable but sterile. These modeling results reflect an extreme scenario characterized by small and isolated populations; more realistic assumptions concerning reproductive rate, sex ratio or gene flow yield extinction risks less dramatic than those predicted by Zayed & Packer [35]. For instance, simulation models have shown that adapted mate choice, with discrimination against diploid males, significantly decreases the risk of extinction [35]. Thus, for species with sl-CSD and unviable or sterile diploid males, the fate of small populations depends on the survival and reproductive behavior of diploid males and the ability of females to discriminate between diploid and haploid males.

We report here a series of experiments assessing the fitness of diploid males in a parasitoid wasp with sl‐CSD, *Venturia canescens*. We show that, in *V. canescens*, diploid males are similar to haploid males for most of the morphological, life‐history and behavioral traits considered. Nevertheless, diploid males take longer to mate in individual tests, and therefore have a lower mating success when competing with haploid males. We also discovered a situation different from the principal scenarios hypothesized by Zayed & Packer [32]: females of *V. canescens* inseminated by diploid males did not produce fewer offspring, but they produced only sons. At the population level, we show through simulation that the likelihood of population extinction is greatly affected by the mating success of diploid males. Our findings thus illustrate how the genetics of small populations can lead to individuals having different types of behavior, and how these types of behavior then affect population dynamics, with consequences potentially as serious as population extinction.

## Methods

### Biological system

*Venturia canescens* Gravenhorst (Hymenoptera: Ichneumonidae) is a parasitoid of pyralid moths, including *Ephestia kuehniella*, *Plodia interpunctella* and *Ectomyelois ceratoniae* [36]. The immature stages of the parasitoid develop on moth larvae within desiccated fruits, such as carobs, dates, medlars and figs, and in husks from nuts and almonds [36]. Adults can live for several weeks, during which time males search for females and females search for hosts [37,38]. The mating system of *V. canescens* has many attributes of panmixis. Both the number of pyralid larvae per fruit and the proportion of hosts parasitized are low [39]. This implies that males and females generally emerge from distant sites with no prior experience of sibs and/or mates, and must search for one another actively. Field and laboratory experiments have shown that males use volatile compounds from hosts and females, synergistically, to find females, whereas the females are guided by the volatile compounds emitted by their hosts [37]. There is some avoidance of mating between siblings [40], but within-population genetic structures inferred from the frequency of 10 microsatellite loci in two different populations suggested an absence of departure from Hardy-Weinberg equilibrium (unpublished data). These data are consistent with a mating system in which males actively search for females, using oviposition sites as a point of rendezvous.

Males are as attracted to mated as to virgin females [37], but females mate only once in laboratory conditions. Consequently, mate choice is thought to be a major component of female fitness. Casual observations have revealed that, during encounters, males perform stereotypical courtship behavior [41], during which females can reject them by pushing them away with their hind legs and preventing them from mounting [42]. Female mate-choice in *Venturia canescens* has been clearly demonstrated by the reluctance of females to mate with their brothers, or with genetically unrelated individuals but in the presence of volatile compounds from their brothers [40].

The *V. canescens* individuals used in this study were obtained from a mass-rearing program initiated nine to ten generations before the experiments with about 100 females captured near Nice, on the French Riviera (43°41′18″N, 7°18′10″E, elevation 130 m). Wasps were reared on second-to fifth-instar *E. kuehniella* larvae, which were themselves reared from eggs provided by commercial breeders (Biotop, Livron-sur-Drôme, France). Hosts were fed on organic wheat semolina and parasitoids were fed on honey and water. Both were maintained in plastic boxes (300 × 100 × 100 mm) at 25 ± 1°C and 45 ± 5% relative humidity (RH), with a 16:8 L:D photoperiod. All the males tested were isolated in a glass vial 24 h after emergence and were fed on honey and water.

### General methods

For comparisons of haploid and diploid males, we used sibmating crosses to increase the number of diploid males available. Under sl-CSD and sibmating conditions, half the parents share an allele in common at the *csd* locus (matched mating). As a result, half their fertilized eggs develop into diploid males [27]. Overall, assuming that the females fertilized half their eggs, brother-sister mating would be expected to yield 50% haploid males, 12.5% diploid males and 37.5% diploid females (thus, 20% of the males should be diploids). Families were initiated with randomly chosen males and virgin females freshly emerged from the mass rearing (F0 generation). Each pair was enclosed for 24 h with hosts in a Petri dish. Brothers and sisters from the offspring (the F1 generation) were then similarly paired and enclosed with hosts for oviposition. These males, emerging with females, were necessarily haploid. Males from the F2 generation were then tested in different experiments, and genotyped for determination of their ploidy.

For subsequent experiments, we produced about 750 F2 males from about 150 F1 brother-sister pairs from about 100 parental families (F0). As expected, post-mortem genotyping showed a minority of the F2 males to be diploid (120 diploid males vs. 570 haploid males among the individuals successfully genotyped), resulting in strongly unbalanced designs. A posteriori, we therefore discarded data concerning a number of haploid males and a number of F1 families that produced no diploid males, to obtain more balanced comparisons. During this procedure, we ensured that there were enough replicates for diploid males, by sometimes keeping two haploid and two diploid males from the offspring of the same F1 pair. In this case, both families and males within families were selected at random. Moreover, the same individuals were sometimes used for the measurement of different traits. These manipulations resulted in the use of 26 diploid males and 24 haploid males from 16 F1 pairs for studies of morphology, adult survival and mate-finding, and 31 diploid males and 33 haploid males from 17 F1 pairs for studies of courtship, mating and sperm transfer. In a last experiment in which males were tested in competition, no selection was applied beforehand, and we used a total of 40 diploid and 179 haploid males from 40 different brother-sister F1 pairs.

The ploidy level (*N* versus 2*N*) of tested males was assessed by determining heterozygosity at microsatellite loci, assuming that (i) males heterozygous for at least one locus are diploid, and (ii) the probability of a diploid individual being homozygous at 10 different microsatellite loci, and therefore misclassified, is small and negligible (given the allelic frequencies in the population from which F0 individuals were selected, the probability of falsely classifying a true diploid male as a haploid was estimated at *p* = 0.0023; X. Fauvergue, unpublished data). DNA was extracted from the thorax and abdomen of the males tested (after storage in 96% ethanol at -20°C), with the commercial prepGeM kit (ZyGeM Ltd, Hamilton, New Zealand). Five dinucleotides (VC-001, VC-002, VC-068, VC-092 and VC-094) and five trinucleotides (VC-009, VC-036, VC-060, VC-066, and Vcan071) (unpublished data, except for Vcan071, Mateo Leach, 2009) were amplified in a multiplex PCR. Each reaction volume included 2 μl of DNA, 5 μl of 2 x Qiagen Multiplex PCR kit (a buffer containing nucleotides and HotStart *Taq* DNA polymerase) and forward and reverse primers at a final concentration of 0.1 μM for markers VC-009, VC-036, VC-068 and VC-092, 0.2 μM for markers VC-060, VC-066 and Vcan071, 0.4 μM for marker VC-094 and 0.6 μM for markers VC-001, VC-002. Forward primers were labeled with one of four different fluorochroms. The reaction volume was adjusted with ultrapure water. The PCR program was as follows: 15 minutes at 95°C, followed by 25 cycles of 30 seconds at 94°C, 90 seconds at 58°C and 60 seconds at 72°C and a final extension at 60°C for 30 minutes. PCR products were then added to 8.75 μl of Hi-Di formamide and 0.25 μl of GeneScan 500 LIZ Size Standards (Applied Biosystems Inc.) and run on an ABI 3130 sequencer (Applied Biosystems Inc.). Sample genotypes were scored with the GeneMarker program (version 1.75 SoftGenetics LLC, USA).

Behavior was observed with a tablet running an event recorder (The Observer, version 10.0; Noldus Information Technology, Wageningen, the Netherlands).

Statistical analyses of the data were based on generalized linear models (GLM) unless otherwise stated. GLMs were implemented with different distributions, depending on the data, and were fitted by maximum likelihood estimation of the parameters associated with ploidy level as a recurrent explanatory variable. Likelihood-ratio tests were used to determine the significance of deviations of these parameters from the expected χ^2^ distributions. Data analyses were performed in the *R* statistical package (*R* Development Core Team 2011).

### Morphometry

Body size is generally correlated with fitness components, including male ability to find females [43-45]. In the context of sl-CSD, diploid males could be larger than haploid counterparts [46], and possibly fitter. We therefore compared the body size of haploid and diploid males. In *V. canescens,* as in most other insects, tibia length is correlated with other morphometric measurements [47] and *in fine*, with male mating success [43-45]. We therefore used hind tibia length as a proxy. Right and left hind tibias were measured three times under a microscope, at × 4 magnification, and the mean of these three measurements was used in subsequent statistical analyses. Male symmetry was estimated by determining the relative difference between left and right tibia lengths (the absolute value of the difference divided by left-right mean size). This index is zero in symmetric individuals, and departs from zero as asymmetry between the left and right sides increases. Male size and symmetry were analyzed with a GLM, using a gamma distribution and an inverse link function.

### Adult survival

We estimated adult lifespan, by recording the dates of male emergence and death, with a precision of 12 h (two daily observations). Between emergence and death, males were enclosed in a 70 × 10 mm plastic tube closed with a piece of cotton wool soaked in water at the top and a piece of cotton wool soaked in honey at the bottom. The amounts of water and honey available to the insects were checked daily and more was added if necessary. The males for which adult lifespan was assessed had been tested in a wind tunnel just after emergence (see below). Flight represents a significant expenditure of energy [48,49] that could affect longevity. We therefore standardized the analysis of adult lifespan by selecting only males that had flown in both assays (i.e., 97% of tested males). We used a GLM with errors following a gamma distribution and an inverse link function to analyze adult lifespan.

### Mate-finding

We assessed the orientation of males towards females in a wind tunnel (described by [37,50]). The males were released 60 cm downwind from a source of volatile sex attractants and their upwind flight was characterized. The source of the volatile compounds consisted of five one-day old virgin females of *V. canescens* and 40 larvae of *E. kuehniella* placed in an open tube (5 cm diameter) placed horizontally, through which air was pushed with an additional pump. The flight chamber of the wind tunnel had dimensions of 150 × 50 × 70 cm, and the release platform was placed 25 cm above the floor of the chamber. Within the flight chamber, light intensity was 4600 lux, there was an airflow of 22 cm.s^−1^, a temperature 25°C and a RH of 45 ± 5%. In addition, visual landmarks for takeoff and in-flight orientation [51] were provided in the form of a false (made from card) plant onto which males were released, two similar false plants placed beside the source, and pieces of colored paper randomly arranged on the four faces of the flight chamber.

Immediately before testing in the wind tunnel, the males were placed for two hours in a large rearing cage within the flight chamber, in which they could practice flying. After this training period, the cage was removed and the test involved the release of each male individually on the takeoff “plant” in two consecutive trials. Male behavior was recorded in real time, from release to landing, or until 5 minutes after release, whichever occurred was the shortest. The source was considered to have been reached if males landed on the tube or flew for more than 5 s less than 5 cm away from the tube, in at least one of the two trials. The females and hosts used as sources of volatile compounds were replaced daily, the tube was cleaned, and the room containing the wind tunnel was thoroughly aired.

On each of 18 consecutive days, 10 to 25 males were tested between 11:30 am and 04:30 pm. The time to takeoff and the duration of flight before landing on the source were calculated from the raw recording and were analyzed for the first successful flight only. The proportion of males reaching the source was also analyzed. Males that did not fly during the two trials, or that flew only once and missed the source, were excluded from the analysis.

Time to takeoff and flight duration (for males that reached the source) were analyzed with a GLM with a gamma distribution of errors and an inverse link function. The proportion of males reaching the source was determined by logistic regression, with a quasi-binomial distribution to correct for overdispersion [52] and a logit link function.

### Courtship, mating and sperm transfer

We investigated the ability of males to court and copulate with females and to fertilize female gametes in a simple set-up. In each test, a male and a genetically unrelated female were placed in a plastic tube (70 × 30 mm) and sexual behavior was observed for 15 minutes or until mating had occurred, whichever occurred first. This made it possible to estimate the following variables: time before first courtship, mean courtship duration (for the various courtship sequences), time to mating, duration of copulation, mating success, and the number of rejections of males by females.

After behavioral observations, each pair of insects was kept in a Petri dish for 24 h under the same laboratory conditions, with food and water, to promote mating, if mating had not occurred during the 15 minutes of direct observation. Each female was then allowed to lay eggs, for four hours, on a patch of 40 second-instar *E. kuehniella* larvae and rearing medium. The females were then dissected in insect Ringer solution, under a microscope, at × 40 magnification. We assessed the success/failure of sperm transfer by determining whether spermatozoa were present in the spermatheca and whether there were females among the offspring. Each tested male was thus characterized in terms of its ability to court and mate with a female within a 15-minute encounter, its ability to transfer sperm, and its ability to sire female offspring.

On each of 17 consecutive days, we tested 10 to 15 pairs between 11:30 am and 04:30 pm. We also subjected 17 one-day-old virgin females from the mass-rearing to the same treatment (including spermathecal inspection), but without mate encounter. These virgin females were used to assess the effect of mating on the production of offspring.

Courtship duration, time to first courtship, time to mating, and copulation duration were analyzed with GLMs with an inverse link function and errors following a gamma distribution. Mating success was evaluated by logistic regression analysis, with a quasi-binomial distribution and a logit link function. We analyzed the number of rejections of males by females, with a GLM with a quasi-Poisson distribution of errors and a log link function. Sperm transfer was analyzed by logistic regression analysis with a quasi-binomial distribution of errors and a logit link function. The number of daughters sired (a measurement of fitness for males), and the total number of offspring were analyzed with a GLM with a quasi-Poisson distribution of errors and a log link function.

### Male mating success in competition

The mating success of diploid males may depend on the intensity of competition with other males, especially haploid males. In addition to the study of reproductive success, we therefore carried out observations in population cages, in which more space was available and females had a greater choice of different males. The males tested were less than three days old and were fed on honey and water.

Mating was observed in a 21 × 31 × 45 cm Perspex cage, between 11:00 am and 3:00 pm, at 25 ± 1°C and a RH of 60 ± 10%. On the day before testing, 16 to 20 virgin males were anesthetized with CO2, and each male was marked with a unique combination of two water-soluble paint dots. Preliminary experiments, in which 59 females were each exposed to five marked and five unmarked males showed mating success to be similar for marked and unmarked males. We can therefore conclude that marking had no effect on mating behavior in *V. canescens*, as also shown in an independent study on female foraging behavior [38]. The 16 to 20 males were then placed together in the observation cage until the start of the test. In the trial, a one-day-old virgin female was introduced into the cage and was observed until mating was observed or until 15 minutes had elapsed, whichever occurred first. When mating occurred, the female was recaptured and the successful male was identified. Each day, we tested 10 females with the same male mating pool, and the procedure was replicated on 13 different days (each day with a different male mating pool).

The ploidy of each male in each pool, regardless of whether it had mated, was determined from individual genotypes. This yielded an estimate of the proportion of diploid males in each male mating pool to which females had been exposed. For each female, mating success and time to mating were assessed by direct observation and the ploidy of the successful partner was determined by genotyping. We determined whether spermatozoa were present in the spermatheca by dissection.

## Results

Analyses of the morphometric, life-history, and behavioral traits of *V. canescens* showed that, in most respects, diploid and haploid males were similar. Diploid males were nonetheless slightly less successful at mating with female than haploid males, and, unlike haploid males, they sired no offspring.

### Differences between male individuals

Adult size, which was determined by measuring hind tibia length, was similar in haploid and diploid males (mean ± SEM: 1435 ± 28 μm and 1462 ± 28 μm respectively; GLM, χ^2^ = 0.005, *df* = 1, *p* = 0.49). Our index of symmetry, based on the relative difference between left and right hind tibia length, was also similar in the two types of males (0.01 ± 0.001 on average; GLM, χ^2^ = 0.02, *df* = 1, *p* = 0.83). Diploid males lived for 16 ± 2.4 days (mean ± SEM), this lifespan being similar to that of haploids (18 ± 1.9 days; GLM, χ^2^ = 0.09, *df* = 1, *p* = 0.68).

Male orientation toward the odors of hosts and females was similar for haploid and diploid males. Similar proportions of diploid (18/31, 58%) and haploid males (23/33, 70%) reached the odor source in the wind tunnel (GLM, χ^2^ = 0.94, *df* = 1, *p* = 0.34). Male ploidy did not influence the time spent elapsed before takeoff from the release point (4.8 ± 1.2 s for diploid males vs. 6.5 ± 3.6 s for haploid males; GLM, χ^2^ = 1.43, *df* = 1, *p* = 0.63). Ploidy also had no effect on flight duration (9.4 ± 3.1 s for diploids and 7.8 ± 1.2 s for haploids, respectively; GLM, χ^2^ = 0.35, *df* = 1, *p* = 0.58).

During the 15 minutes in which courtship behavior was directly observed, 81% of diploid males (21/26) and 96% of haploid males (23/24) engaged in courtship (Chi-squared test, χ^2^ = 1.45, *df* = 1, *p* = 0.23). Neither mean courtship time nor the time elapsed before first courtship (*i.e.* time to courting) differed significantly between haploid and diploid males (Table 1). Overall, one third of the tested males successfully mated with the female during the 15 minutes of observation and, despite a tendency towards lower mating success for diploids than for haploids (19% vs. 38%), this difference was not statistically significant (Table 1). Females rejected haploid and diploid males with a similar frequency (Table 1). The main difference between haploid and diploid males was the time elapsed before mating (Table 1). It took diploid males four times longer than haploid males to achieve successful mating.

**Table 1:** **Mating behavior of** ***Venturia canescens*** **males as a function of ploidy (diploid or haploid)**

	**Diploid**	**Haploid**	**χ** ^**2**^	***p*** **-value**
Time to 1st courtship (s)	40 ± 16	45 ± 18	0.20	0.82
Courtship duration (s)	12 ± 3	11 ± 2	0.07	0.79
Time to mating (s)	302 ± 99	71 ± 11	7.13	<0.001
Copulation duration (s)	170 ± 81	110 ± 19	0.63	0.29
Rejected males	19% (5/26)	29% (7/24)	0.68	0.42
Mating success	19% (5/26)	38% (9/24)	2.08	0.15

Mean values ± standard errors, or proportions, are given for each behavioral item. χ^2^ and *p*-values were obtained in likelihood-ratio tests for the effect of male ploidy (one degree of freedom) derived from generalized linear models fitted to the data.

**Figure 1:**
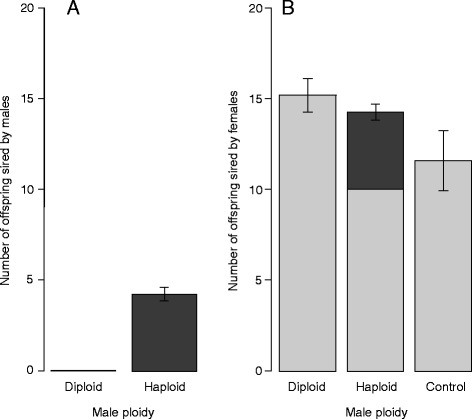
Fitness of diploid versus haploid males, and consequences for offspring sex ratio. **A**. Mean numbers of offspring sired by diploid and haploid males; **B**. Mean number of offspring produced by females that had mated with diploid or haploid males or had not mated (control). Light gray bars correspond to sons and dark gray bars correspond to daughters. Error bars indicate the standard error of the mean number of offspring.

Both diploid and haploid males transferred sperm to females, as demonstrated by the presence of sperm in the spermatheca of females that had been exposed to males. After 24 h, 41% of diploid males (7/17) and 61% of haploid males (14/23) had mated with females and transferred sperm, and these proportions were not significantly different (GLM, χ^2^ = 1.53, *df* = 1, *p* = 0.23). However, unlike haploid males, diploid males sired no offspring: when exposed to 40 hosts for 2 h, females that had mated with a diploid male produced no daughters, whereas females that had mated with a haploid male produced a mean of five daughters (Figure 1A). However, we identified one exception: the presence of one triploid daughter in the progeny of a female that mated with a diploid male. All the males sired by mated females were haploid, whether the male mating partner was haploid (subsample, *N* = 129) or diploid (subsample, *N* = 159). Thus, despite the similarities between diploid and haploid males for all the traits measured, apart from the difference in time to mating in individual tests, the diploid males were unable to produce offspring and were therefore not fit.

### Male mating success in competition

**Figure 2:**
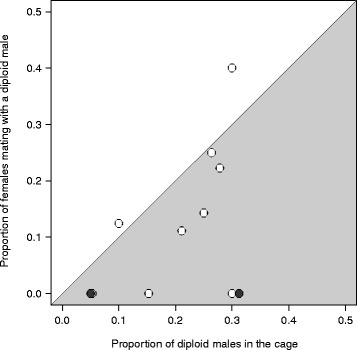
Diploid versus haploid male mating success in competition. Proportion of females that mated with a diploid male versus the proportion of diploid males present in the population cages (13 replicates). Each dot represents one (white dots) or two replicates (black dots). The light gray area indicates replicates for which the proportion of females mating with a diploid male is lower than the proportion of diploid males in the cage.

If mating were random with respect to male ploidy, we would expect the proportion of females mating with a diploid male to be similar to the proportion of diploid males within the mating cage. However, this was not we observed. When pooled across all replicates (the 13 cages), the proportion of females mating with a diploid male (0.10; 9/89) was about half the proportion of diploid males present in the cages (0.19; 47/247; Fisher’s exact test, *p* = 0.034). Hence, in conditions of competition, diploid males had a lower mating success than haploid males. Without pooling over replicates, we used a pairwise Wilcoxon-Mann–Whitney test to compare the proportion of females mating with a diploid male to the proportion of diploid males within a given cage (*N* = 13). Again, we found a significant difference (*V* = 10, *p* = 0.014). We also found that the probability of mating with a diploid male was lower than the relative abundance of diploid males in 11 of the 13 replicates (Figure 2). Such a result would be unlikely if haploid and diploid males had a similar mating success (the value of 11/13 obtained has a probability *p* = 0.011, assuming a binomial distribution and an expectation of 0.5).

A non-negligible number of females (36 of 129, corresponding to 30%) did not mate within the 15-minute observation period, but the probability of mating was unaffected by the proportion of diploid males present in the cage (mixed GLM with a binomial distribution of errors and a logit link function, and replicate as a random effect: χ^2^ = 0.80, *df* = 1, *p* = 0.37). The time elapsed before mating was not affected by the proportion of diploid males in the cage (mixed GLM on log-transformed durations, with a Gaussian distribution of errors and identity link function: χ^2^ = 0.37, *df* = 1, *p* = 0.54) or by the ploidy of the successful male (χ^2^ = 1.58, *df* = 1, *p* = 0.21).

### Modeling of consequences for the population

Our mating experiment revealed that females that had mated with a haploid male or with a diploid male, and females that had not mated at all, produced a similar total number of progeny (GLM, χ^2^ = 9.9, *df* = 2, *p* = 0.14, Figure 1B). However, these groups of females differed in terms of the sex ratio of their offspring. Females that mated with a haploid male produced some daughters, whereas virgin females and females that mated with a diploid male produced no daughters but a similar number of sons (15 and 12 sons, respectively, in four hours of exposure to hosts; GLM contrast matrix, χ^2^ = 35.77, *df* = 2, *p* = 0.09). Hence, from the female perspective, mating with a diploid male has the same fitness consequence as not mating at all. The production of an all-male progeny by mated females is referred to as “pseudovirginity” [53].

Zayed and Packer [32] predicted the probability of extinction for populations of haplodiploid organisms with sl-CSD, random mating, environmental and demographic stochasticity. In their model, two contrasting scenarios for the fitness of diploid males were considered: (1) inviability and (2) sterility, resulting in the death of all eggs fertilized by sperm from diploid males (*i.e.*, mortality of potential triploid daughters). If viable, diploid males were assumed to be as successful as haploid males at encountering and mating with females. Our results for *V. canescens* are thus inconsistent with the assumptions of the model developed by Zayed and Packer [32].

We therefore developed a stochastic individual-based model, assuming pseudovirginity and various rates of diploid male mating success. This approach has a two-fold rationale: first, a model fitting the behavior of *V. canescens* should help to unravel the demographic consequences of the behavior observed in this species, improving our understanding of the relevance of this behavior at the population level. Second, our model aims to extend the framework first developed by Zayed and Packer [32]. This is of interest because, in several other species with sl-CSD, diploid males have been shown to have a mating success that is neither negligible nor as high as that of haploid males [29,54-56]. In addition, diploid males trigger pseudovirginity in several species, suggesting that the real-life scenario may not have been taken into account in the model developed by Zayed & Packer [32]. We thus modified the model framework described by Zayed & Packer [32], to make it possible to compare a scenario in which viable diploid males sire no offspring (pseudovirginity) with a scenario in which viable diploid males sire inviable triploid females (mortality of potential daughters). We also assumed that diploid males might be less successful than haploid males at finding and mating with females. We ran sensitivity analyses to investigate the consequences of diploid male mating success for population dynamics.

Our model is described in detail in Additional file 1. For each scenario and various values of mating probability for diploid males, 1000 simulations were run, each across 100 generations. Simulations were replicated with randomly drawn values for the parameters environment carrying capacity (*K*) and net reproductive output (*NRO*, the expected number of offspring per female). The probability of extinction P(*E*) was estimated as the proportion of simulations ending in population extinction. We investigated the processes leading to extinction, by also estimating the proportion of males in the population and population growth. For population growth, we calculated the proportion of pairs of subsequent generations for which the population increased or stayed the same. This proxy for population growth rate is the best descriptor of the dynamics of small populations [57,58].

**Figure 3:**
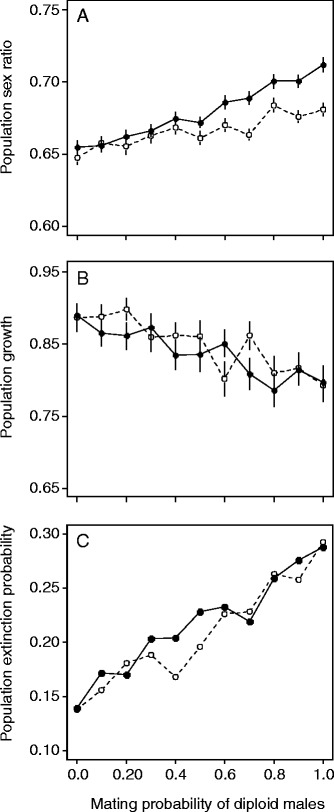
Population dynamics of hymenopterans with diploid males. Effect of diploid male mating success, relative to that of haploid males, on the population sex ratio (proportion of males), population growth rate and extinction probability. Two alternative scenarios were investigated: females mating with diploid males produce inviable triploid offspring (open circles, dotted lines) or are pseudovirgins (closed circles and solid lines). Simulations were run with randomly drawn values for the parameters environment carrying capacity (*K*) and female net reproductive output (*NRO*, the expected number of offspring produced per female), with 50 ≤ *K* ≤ 500 and 2 ≤ *NRO* ≤ 10. Means and standard errors, estimated on 100 replicates, are displayed for population sex ratio **(A)** and population growth **(B)**. The probability of population extinction **(C)** was estimated from 1000 replicates.

Simulations showed that the proportion of males in the population increased with increasing diploid male mating success (Figure 3A). Unsurprisingly, the bias in sex ratio was strongest for the scenario in which diploid males triggered pseudovirginity. Greater mating success of diploid males was also associated with a decrease in population growth (Figure 3B) and an increase in the probability of population extinction (Figure 3C). This effect on the likelihood of extinction was strong, with extinction probability increasing from 0.15 to 0.30.

## Discussion

Single-locus complementary sex determination in insects of the order Hymenoptera results in a severe form of inbreeding depression underpinned by overdominance: individuals homozygous for the *csd* gene develop into diploid males that are mostly inviable or sterile [28,29,59]. Diploid males are thus a reproductive dead end. Nevertheless, subtle changes in some of their fitness components may have drastic consequences for the evolution of female behavioral responses to prevent the production of, or mating with, diploid males, as well as for the dynamics of small populations. We discuss our findings in light of these evolutionary and demographic aspects.

In *Venturia canescens*, diploid males resemble haploid males in most respects. Despite the diverse morphological, behavioral, and life-history traits analyzed, we found only two significant differences between diploid and haploid males. First, for single males enclosed with single virgin females, diploid males took four times longer to mate than haploid males. A similar delay in mating has also been reported in other species, such as *Cotesia rubecula* [54]. Consistent with this finding, diploid males had a lower mating success than haploid males in situations in which diploid and haploid males competed for females and females had the opportunity to choose their own mate. It is unclear whether these differences result from discrimination against diploid males by females, or from independent attributes of diploid males. The similar probability of rejection by females for diploid and haploid males in the no-choice experiment suggests that the lower mating success of diploid males in the choice experiment is not a consequence of active female discrimination. Our results therefore suggest that in *V. canescens*, diploid males are less fit than haploid counterparts. This appears surprising because diploid and haploid males were of similar size and symmetry and, in wind tunnel experiments, they took off and flew as rapidly as haploid males towards females. Consistently, similar phenotypes have been observed for the haploid and diploid males of several other parasitoid species [55,59].

Our approach suggests that even if the effects of ploidy are not revealed when selected phenotypic traits are considered independently, they may become much more evident in more realistic experimental set-ups in which males compete for females and the response investigated integrates all traits. It may be argued that sequential mate-encounter is the rule in species like *Venturia canescens* where males search actively and individually for pheromone-emitting females [37] and hence, do not form mating swarms. For such species, exposing females to a group of competing males may not reflect a truly natural situation. Nonetheless, placing haploid and diploid males in large cages does not necessarily imply female mate-choice typical of lek mating systems. Rather, by integrating the whole suite of behaviors involved in mate finding, it may exacerbate differences between male types, which are not observed when each trait is analyzed separately.

The second difference between diploid and haploid males concerned their fertilization success. Both haploid and diploid males transferred apparently viable sperm to the female sperm storage organ (the spermatheca), but none of the female oocytes were fertilized by sperm from diploid males. Thus, in *V. canescens*, diploid males are viable but sterile and have a lower mating success than haploid males.

Whether diploid males are inviable, or viable but sterile, for the parents, each diploid male produced is equivalent to the death of a female offspring. This major fitness cost has favored the evolution of inbreeding avoidance through a premating refractory period [60], natal dispersal [61,62], possibly male-biased and combined with protandry [63], or active mate choice [40,60,64]. However, there are exceptions: the diploid males of some species are fully or partially fertile [29,30,55,56] and may, therefore, not trigger inbreeding avoidance [65]. In *V. canescens*, females use volatile compounds to avoid mating with genetically related individuals [40]. Given the negligible level of inbreeding depression for other morphological and life-history traits [66], inbreeding avoidance in *V. canescens* can best be interpreted as an adaptive response designed to prevent the production of sterile diploid males.

Four different scenarios are generally described for diploid males according to their mating and fertilization success [28,59]: (1) Diploid males do not mate with females (e.g. [67]); (2) diploid males mate with females, but the inability of unreduced diploid sperm to fertilize female oocytes imposes constraints on females mating with diploid males. These females produce as many offspring as mated females, but only males (e.g. [68]); (3) diploid males mate with females, and diploid spermatozoa fertilize oocytes, generating triploid female offspring that are themselves inviable or sterile (e.g. [55]); (4) diploid males mate with female and sire normal, diploid daughters (e.g. [29]). Our findings for V. canescens suggest that both the first and second scenarios apply in this species. Diploid males are as viable as haploids and can mate with females, albeit less successfully than haploid males. They are sterile, as shown by the male-only reproductive output of the females they inseminate. Consequently, in V. canescens, females mating with diploid males are considered to be pseudovirgins [53], as they produce as many offspring as mated females, but only males.

In species such as *V. canescens*, the costs of mating with a diploid male should be analyzed in the theoretical framework of constrained oviposition and sex allocation [53]. In large, randomly mating populations at sex ratio equilibrium, sons and daughters are strictly equivalent for the parents, in terms of their ability to generate grandchildren [69]. In such panmictic populations, mating with a diploid male and reproducing like a virgin have no cost. In the wild, populations of *V. canescens* resemble panmictic populations. First, as a consequence of host dispersion and low rates of parasitism, most males and females emerge on different patches that may be some distance apart [39,70], and males must search actively for females, using a synergic mixture of semiochemicals from females and hosts [37]. Second, genetic analyses of two field populations obtained from sites some distance apart in southeastern France and based on 19 microsatellite markers suggested an absence of strong departure from Hardy-Weinberg equilibrium (unpublished data). Third, secondary sex ratios are weakly biased toward females, as a possible evolutionary response to sustained constrained oviposition [71], with the possible consequence of maintaining the population sex ratio at Fisherian equilibrium. In such panmictic populations, females mating with diploid males and producing only sons should have a fitness similar to that of females mating with a haploid male and producing both sons and daughters.

However, the conclusion that mating with a diploid male has no cost may not apply to small or isolated populations because, in such populations, significant fluctuations of the sex ratio equilibrium favor mixed-sex broods [72,73]. Consequently, pseudovirginity, resulting from mating with a diploid male, becomes costly in precisely the situation in which diploid males would be expected to be more frequent. Over evolutionary timescales, selection for female discrimination against diploid males is therefore likely to depend on the frequency of population bottlenecks and concomitant variations of the proportion of diploid males. Given the scarcity of robust data concerning the proportion of diploid males in the field [28], a more detailed discussion of adaptive responses would be speculative.

By contrast, the ability of sterile diploid males to mate with females may have more serious effects when seen in the context of population dynamics. The probability of population extinction is much higher if sterile diploid males are viable and capable of mating, because, in this case, the decrease in net reproductive rate spans over two generations [32,74]. Our simulation model assumes that diploid males have variable mating success. Regardless of whether mating with a diploid male triggers the death of fertilized eggs or pseudovirginity, the same relationship between diploid male mating success and extinction probability applies: increasing mating success of diploid males is associated with an increase in the proportion of males in the population and a decrease in growth rate, resulting in a monotonous and linear increase in the probability of population extinction.

## Conclusion

Our study shows that individual behavior, such as the ability of males to mate or female mate choice, may have serious consequences for the demography of small populations. Linking individual behavior to population dynamics is an exciting avenue of research [75] to which host-parasitoid models have made a substantial contribution [76-81]. Nevertheless, most previous studies have focused on types of behavior promoting negative density-dependence and population stability. Our model contrasts with these classic studies in that, rather than trying to identify processes improving demographic stability (the Holy Grail of host-parasitoid population dynamics; [79]), we focused on the small-population paradigm [1] and non-equilibrium ecology [82], by relating individual behavior to population extinction. We also included key genetic processes governing small populations, such as inbreeding depression. This focus on instability opens up new possibilities in population biology and management [6,35,83,84].

## Additional file

Additional file 1:
**Details for the population model.**
