# Supplementary material for: Sterile males in a parasitoid wasp with complementary sex determination: from fitness costs to population extinction
Source: BMC Ecol. 2015 May 12;15:13. doi: 10.1186/s12898-014-0032-6 (PMC4449571; doi:10.1186/s12898-014-0032-6)
Supplement: Additional file 1: — Details for the population model. [file 12898_2014_32_MOESM1_ESM.pdf]

Additional File 1

We developed a simulation model for analysis of the impact of variations in the mating success of diploid males triggering pseudovirginity on the extinction probability of hymenopteran populations with single-locus complementary sex determination (sl-CSD). Our model is derived from that of Zayed & Packer [1], particularly from the case in which diploid males produce inviable triploid offspring. We added a novel scenario to this model, in which diploid males induce pseudovirginity in the females with which they mate, as observed in *Venturia canescens* and several other species with sl-CSD. We also assumed that the mating probability *p* (relative to haploid males) of diploid males could be lower than 1, either because these males are less efficient than their haploid counterparts at finding females, or because they are discriminated against by the females they encounter. In this population model, as assumed by Zayed & Packer [1], females always mate, regardless of population density. There is therefore no mate-finding Allee effect, so a male-biased sex ratio results solely from mating with diploid males.

In each simulation run, the population had an initial number of individuals, *N*, set at the environment carrying capacity, *K*, and a balanced 0.5 sex ratio, as observed in *V. canescens* (Metzger, 2008). The initial number of alleles at the CSD locus, *n*, was determined from the effective population size, *Ne*, and the mutation rate, *μ*, as described by Cornuet [2]:

$$n = \sqrt{\frac{-2N_e}{\log\left(\mu\sqrt{8\pi}N_e\right)}}$$

Ne was defined as a set fraction of initial population size (*Ne* = 0.2 *K*), and *μ* was set at 10^{-6} mutations per generation. The *n* alleles at the *csd* locus were randomly assigned to each of the *K* founder individuals, assuming that the *K*/2 diploid individuals were heterozygous and, therefore, female. At each generation, the following procedure was applied: (1) males and females were paired at random, with the assumption that females always mate (i.e., no mate-finding Allee effect), but only once, whereas males may mate with several females. Diploid males have a relative mating probability of *p*, varying between 0 (diploid males never mate) and 1 (the probability of mating is identical for diploid and haploid males). (2) For each female, the number of offspring depended on a constant expected net reproductive output (*NRO*), to which we applied variation between generations (environmental stochasticity) and between individuals (demographic stochasticity). For each female, the number of offspring was drawn from a

normal distribution with mean *Xe* and variance *Vd* = 6 *NRO*, *Xe* being itself drawn at each generation from a normal distribution with mean *NRO* and variance *Ve* = *NRO*. (3) Each offspring could develop from a fertilized or unfertilized egg, fertilization status being drawn from a binomial distribution with a mean of 0.5. (4) For each offspring, *csd* alleles were assigned in accordance with standard Mendelian inheritance. (5) The development of the offspring into a male or female depended on fertilization status, alleles at the *csd* locus, and the hypothetical scenario. (6) If the total number of offspring exceeded *K*, supernumerary individuals were eliminated at random. (7) The offspring and their genotypes replaced their parents and parental genotypes, and the loop was run again until the 100th generation.

One thousand replicated simulations were run for each of the two scenarios and values of *p* from 0 to 1, with a step size of 0.1. For each run, we estimated three output variables: (1) extinction (absence of individuals before the 100th generation); (2) mean population sex ratio (proportion of males); (3) the rate of population increase, calculated as the proportion of pairs of successive time steps for which the number of individuals increased or had reached the carrying capacity. *K* and *NRO* have a strong impact on the effect of sl-CSD on population dynamics [1]. Rather than carrying out sensitivity analyses for the combined effects of *p*, *K* and *NRO*, or using arbitrary values for *K* and *NRO*, each simulation was run with values of *K* and *NRO* drawn at random from the range first used by Zayed & Packer [1]: $50 \leq K \leq 500$ and $2 \leq NRO \leq 10$. As sl-CSD has no impact on population dynamics over a wide range of values in this range, any effect of diploid male mating success observed, even if of relatively low magnitude, is likely to be highly significant.

References

1. Zayed A, Packer L: **Complementary sex determination substantially increases extinction proneness of haplodiploid populations**. *Proceedings of the National Academy of Sciences of the United States of America* 2005, **102**(30):10742-10746.
2. Cornuet JM: **Rapid estimation of the number of sex alleles in panmictic honeybee populations**. *Journal of Apicultural Research* 1980, **19**(1):3-5.
